# The impact of digital financial inclusion and urbanization on agricultural mechanization: Evidence from counties of China

**DOI:** 10.1371/journal.pone.0293910

**Published:** 2023-11-02

**Authors:** Cunjing Liu, Lei Chen, Zhezhou Li, Difan Wu

**Affiliations:** 1 School of Economics and Management, Yan’ an University, Yan’ an, China; 2 Rural Development Insitute, Yan’ an University, Yan’ an, China; 3 School of Economics and Management, Fuzhou University of International Studies and Trade, Fuzhou, China; 4 State Grid Shanghai Electric Power Company Shibei Power Supply Company, Shanghai, China; Hamdard University, PAKISTAN

## Abstract

This paper expounds the theoretical logic among digital inclusive finance, urbanization, and agricultural mechanization level, puts forward the research hypothesis, and then selects the county unbalanced panel data of 1309 counties in China from 2014 to 2020 based on the two-way fixed model with standard error clustering to county level and mediating effect model for empirical data regression analysis. Through baseline regression analysis, mediation effect analysis, and heterogeneity analysis, the findings of this paper are as follows. First, digital financial inclusion has a significant positive effect on the growth of agricultural mechanization. Second, digital inclusive finance at the county level can also indirectly affect the growth of agricultural mechanization through urbanization. That is, agricultural mechanization has an intermediary effect between the financial agglomeration at the county level and the growth of farmers’ income. Third, the impact of county-level digital financial inclusion on the growth of agricultural mechanization level is significantly heterogeneous, and the promoting effect is significant in areas with balanced grain production, national-level poor county or contiguous areas of dire poverty, and areas with a good foundation for digital financial inclusion. By analyzing digital inclusive finance, urbanization, and agricultural mechanization, this paper proposes targeted policy recommendations. First, the government can promote agricultural mechanization by developing digital financial inclusion. Second, the government should guide and accelerate the process of digital financial inclusion, promoting urbanization thereby amplifying the positive impact of digital financial inclusion on agricultural mechanization. Third, given the heterogeneity of the impact of digital financial inclusion on agricultural mechanization, local development should focus on developing different dimensions of digital financial inclusion according to specific conditions.

## 1. Introduction

Agricultural mechanization has promoted the transformation of the agricultural development mode and realized the increase in grain production, agricultural efficiency, and farmer income by improving the comprehensive agricultural production capacity and providing equipment support to revitalize rural industries [1]. Currently, the foundation of agricultural mechanization in China is still relatively weak, and the problem of unbalanced and inadequate development is prominent, making it challenging to meet the realistic needs of serving the rural revitalization strategy and supporting the high-quality development of agriculture [2]. In the post-pandemic era, it is a great challenge to improve vulnerable industries and populations [3–5], especially for agriculture and farmers, to which the development of agricultural mechanization is an effective solution.

The development of traditional finance in rural areas faces problems such as "high cost" and "difficult supervision," and service efficiency is relatively low. Digital inclusive finance provides farmers with convenient financial services through the Internet, big data, and other technologies, overcoming the shortcomings of insufficient supply and low efficiency of traditional finance in rural areas and alleviating the problems of "high threshold" and "difficult access" for farmers to participate in the financial market. Digital inclusive finance has effectively improved the level of financial supply and the efficiency of financial services in rural areas and has played an essential role in stabilizing farmers’ production and operation input [2]. Digital inclusive finance can ease the constraints of capital factors in the agricultural sector, thereby improving agricultural mechanization by increasing the input of agricultural capital factors [6].

Although digital financial inclusion has the potential to promote agricultural mechanization, the digital divide and elite capture effect limit its implementation effect in rural areas [7]. The elite capture effect makes accessing digital financial services easier for those with good financial resources, while marginalized farmers and impoverished groups often need equal opportunities [8]. In some regions, especially in rural areas, the penetration rate of digital financial inclusion may be low due to limited infrastructure and technological conditions, resulting in an imbalance in urban and rural financial services [9]. Due to the limited digital infrastructure and technical conditions, in some rural areas, a digital divide makes the penetration rate of digital inclusive finance low, and urban and rural financial services are unbalanced, thus hindering the flow of capital factors to agriculture [10].

There is limited research on digital financial inclusion and agricultural mechanization. Most of the existing literature about the impact of digital financial inclusion in agriculture focuses on increasing farmers’ income [11, 12], improving agricultural production efficiency [13], and improving agricultural output [14] and agricultural supply chains [15]. The few literatures that focus on the impact of digital financial inclusion on agricultural mechanization vary in their focus. Based on the data from Chinese Family Panel Studies(CFPS) in 2014, 2016, and 2018, Zhang and Wang (2021) found that the development of digital inclusive finance promoted the substitution of capital for labor in agricultural production, thus improving agricultural mechanization. This promotion effect gradually weakened with time [16]. Cai et al.(2023) based on panel data from 30 Chinese provinces from 2011 to 2020, the empirical test demonstrates that digital financial inclusion effectively stimulates the upgrading and innovation of agricultural machinery and equipment manufacturing related to agricultural production activities [17]. Yan et al. (2022) based on the digital financial inclusion index and the data from the China Labor-force Dynamics Survey (CLDS) in 2012 and 2014, constructed mixed cross-section data after matching macro data and microdata and studying the impact of the development of digital financial inclusion on agricultural mechanization and its mechanism from the perspective of the development of agricultural machinery operation service market [18]. Sun et al. (2022) based on the data of 1869 counties, the SARAR model study found that developing digital inclusive finance is a critical path to promote the mechanization of agriculture and the spatial spillover effect [2].

There are few studies on digital financial inclusion and agricultural mechanization from the dimension closest to the actual agricultural and farmer situation at the county level. A few studies conducted from the county dimension have chosen the SARAR model that considers the spatial correlation between individuals but requires a strong balance of data, which led to it having to supplement the missing values of some county-level variables with interpolation. This method makes the data imply strong assumptions about the smoothness and linearity of the data, which may cause that to be divorced from the actual situation. Besides, it does not exclude counties with changing administrative divisions from the sample, and the impact of this influential heterogeneous factor on socio-economic and agricultural mechanization cannot be excluded.

This paper discusses the theoretical logic among digital inclusive finance, urbanization, and agricultural mechanization. Then, this paper selects the county unbalanced panel data of 1309 counties in China from 2014 to 2020 and uses the two-way fixed model and mediating effect test model for empirical analysis. Compared with the existing literature, the marginal contribution of this paper is reflected in the following aspects. First, this paper expands the empirical data research on the relationship between digital financial inclusion and agricultural mechanization. Given the weak influence of spatial correlation on agricultural mechanization, the bidirectional fixed model adopted in this paper by clustering standard errors to counties can better deal with the characteristics of China’s county dimensional unbalanced panel data and eliminate the influence of individual and time-invariant factors. Second, this paper attempts to further analyze the mechanism of digital inclusive finance on agricultural mechanization from the perspective of urbanization as a new mediating variable and clarify the relationship between digital inclusive finance, agricultural mechanization, and urbanization. Third, this paper attempts to explore the disequilibrium effect of digital financial inclusion on agricultural mechanization by heterogeneity analysis, which includes the main grain production areas and grain production balance areas, national-level poor county or contiguous areas of dire poverty, and the relative level of development of digital inclusive finance.

## 2. Theoretical analysis

### 2.1. The direct impact of digital financial inclusion on agricultural mechanization

From the micro dimension, digital inclusive finance can alleviate the credit constraints of relatively vulnerable groups to promote farmers’ investment in production and management represented by agricultural machinery. Regarding geographical distance constraints and traditional finance’s exclusion of traditional farmers [19], credit constraints became the main reason hindering agricultural mechanization [20]. The development of digital inclusive finance breaks the spatial restriction between financial demanders and suppliers [7], accurately quantifies the credit level of small-scale financial demanders, reduces financial transaction costs, optimizes the regional financial supply mode [21] and improves the efficiency of traditional finance in serving farmers [22]. Digital inclusive finance provides farmers with convenient financial services through the Internet, big data, and other technologies, overcoming the shortcomings of insufficient supply and low efficiency of traditional finance in rural areas and alleviating the problems of "high threshold" and "difficult access" for farmers to participate in the financial market [9]. Digital inclusive finance has effectively improved the level of financial supply and the efficiency of financial services in rural areas and has played an essential role in stabilizing farmers’ production and operation input [2], represented by special agricultural machinery.

From the macro dimension, digital financial inclusion allows capital elements to pour into the agricultural sector more cheaply, thus improving agricultural mechanization. The lack of capital factors in the agricultural sector limits the improvement of agricultural mechanization level. Improving the level of agricultural mechanization requires the input of capital factors. However, the sources of capital factors in the agricultural sector are few, and they will transfer to the non-agricultural sector in reality [23]. Under the background of digital economy development, digital inclusive finance can not only use digital technology to overcome the pain points of high transaction costs and asymmetric information of traditional rural finance but also greatly alleviate the problems of complicated and expensive financing in the agricultural sector, promote agricultural development [24]. Digital inclusive finance breaks the operation mode of traditional finance, broadens the source channels of capital elements in the agricultural sector, and opens up the "last mile" of financial services in agriculture and rural areas with a low-cost, convenient, and sustainable model [25]. Based on the panel data of provincial level [26], county level [27], and rural household level [28], all found that digital inclusive finance can provide sufficient capital elements for agricultural economic development [27], then effectively promote the level of agricultural mechanization [1].

Based on the above analysis, this paper proposes Hypothesis 1.

**H1:** The development of digital financial inclusion can promote agricultural mechanization.

### 2.2. Digital financial inclusion, urbanization and agricultural mechanization

Digital financial inclusion can promote the process of urbanization to a certain extent. Digital financial inclusion provides more financing channels, financial support, and development opportunities for entrepreneurs and small and micro enterprises [22]. This support can increase entrepreneurship and employment opportunities and attract people to cities. Besides, digital financial inclusion enables more people to access financial services by lowering the accessibility and cost of financial services [29], exerting the multiplier effect of finance to reduce the problem of absolute poverty. Digital financial inclusion can help residents in poor and rural areas more easily integrate into the urbanization process and enjoy the development opportunities brought by urbanization [30].

The inference that the development of digital financial inclusion helps urbanization ignores the following three aspects. There is a financial exclusion problem in agriculture. Since the efficiency of the non-agricultural sector is higher than that of the agricultural sector, and the efficiency of the urban sector is higher than that of the rural sector [31], under the condition of profit-seeking [32], digital inclusive financial institutions will still invest capital elements into the non-agricultural sector and the urban sector. If digital inclusive financial institutions fail to effectively address the issue of excluding traditional financial products from the agricultural and rural sectors, the potential of digital financial inclusion in driving urbanization will be severely limited. Second, although the development of digital inclusive finance can theoretically help the development of the agricultural economy through innovative financial models, the development of digital inclusive finance will not spontaneously promote urbanization in the case of low agricultural productivity and rural labor outflow, that is, the beneficial effect of digital inclusive finance is limited [33]. Moreover, the phenomenon of elite capture in digital financial inclusion amplifies the profit-driven nature of finance. Coupled with the existence of the digital divide, in areas with weak digital foundation [34], especially in rural areas, the development of digital inclusive finance may not only fail to improve the allocation structure of capital factors between urban and rural areas [11], but may lead to new urban-rural differentiation.

Based on the above analysis, this paper proposes Hypothesis 2a.

**H2a:** Digital inclusive finance impacts the level of urbanization development.

The induced technological change theory points out that the market prices of relatively abundant factors are lower, and those of relatively scarce factors are higher due to the difference in resource endowment. Under market prices, technological change always tends to use more abundant elements and save the relatively scarce ones [35]. Urbanization provides diversified employment opportunities and a comfortable working environment, and the wage income of the labor force engaged in the secondary and tertiary industries in the city is significantly higher than that of the general agricultural work [36, 37]. Therefore, based on the relative scarcity of the agricultural labor force, the farmers will actively use machinery to replace the labor force under high relative prices and strong substitution conditions. In addition, with the advancement of urbanization, the rural economy has gradually realized large-scale operation, and the scale of agricultural production has expanded. Agricultural mechanization can improve production efficiency and output and better adapt to the needs of large-scale agricultural production [10].

Based on the above analysis, this paper proposes Hypothesis 2a and Hypothesis 2c.

**H2b:** Urbanization development will improve the level of agricultural mechanization.
**H2c:** Urbanization plays a mediating role in the relationship between digital inclusive finance and agricultural mechanization.

**Fig 1:**
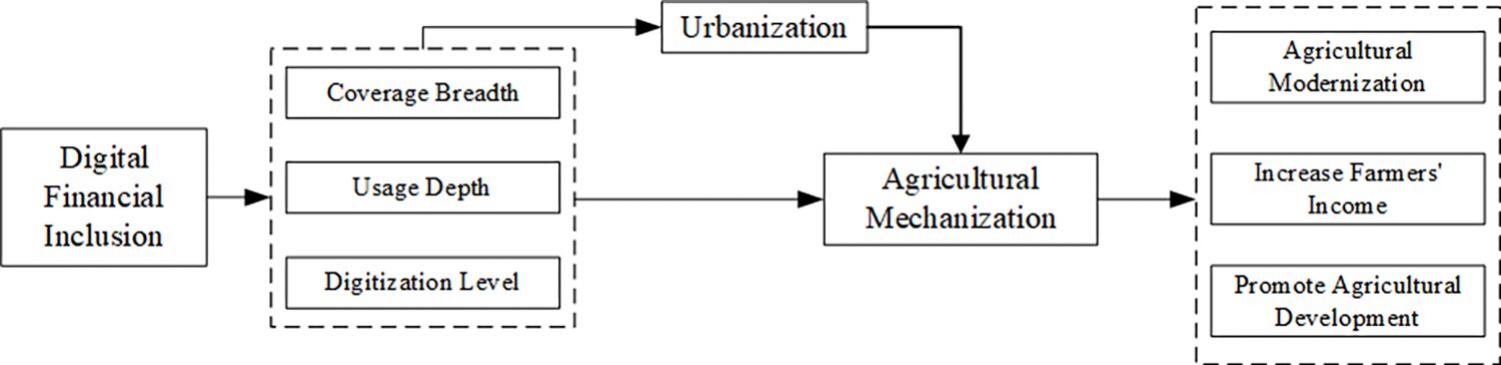
Theoretical mechanism diagram.

According to the above theoretical analysis, the theoretical analysis mechanism diagram of this paper is shown in Fig 1.

## 3. Materials and methods

### 3.1. Data source

This paper uses the Digital Financial Inclusion Index of county-level administration units to represent the development of digital financial inclusion in China. The annual Digital Financial Inclusion Index data of explanatory variables is co-compiled by Peking University’s Research Center for Digital Finance and Ant Financial Services Group. Based on county-level administration units’ data from 2014–2020. Socio-economic data at the county dimension comes from the China County Statistical Yearbook (2014–2020) and the China Stock Market & Accounting Research Database(CSMAR). National-level Poor County data come from China National Rural Revitalization Bureau. Contiguous Areas of Dire Poverty data comes from an Explanation by the Poverty Alleviation Office of the State Council on the Announcement of the List of Counties and Districts in China’s Continuously Impoverished Areas. Major grain-producing areas, grain balance areas, and main grain sales areas data come from China’s Grain Production Capacity Planning (2009–2020). The nighttime lighting data processing method uses the "pseudo-invariant pixel method" to calibrate Defense Meteorological Satellite Program/Operational Linescan System(DMSP-OLS) data. It takes into account the consistency of time resolution between DMSP-OLS data and National Polar-Orbiting Partnership’s Visible Infrared Imaging Radiometer Suite(NPP-VIIRS) data as the missing data in the original monthly data is repaired before synthesizing annual SNPP-VIIRS data. After excluding the counties with changes in jurisdictions of administrative units and serious missing data, unbalanced panel data from 2014 to 2020 were obtained for 1309 counties in China.

### 3.2. Definition of variables

#### 3.2.1. Agricultural mechanization level

**Fig 2:**
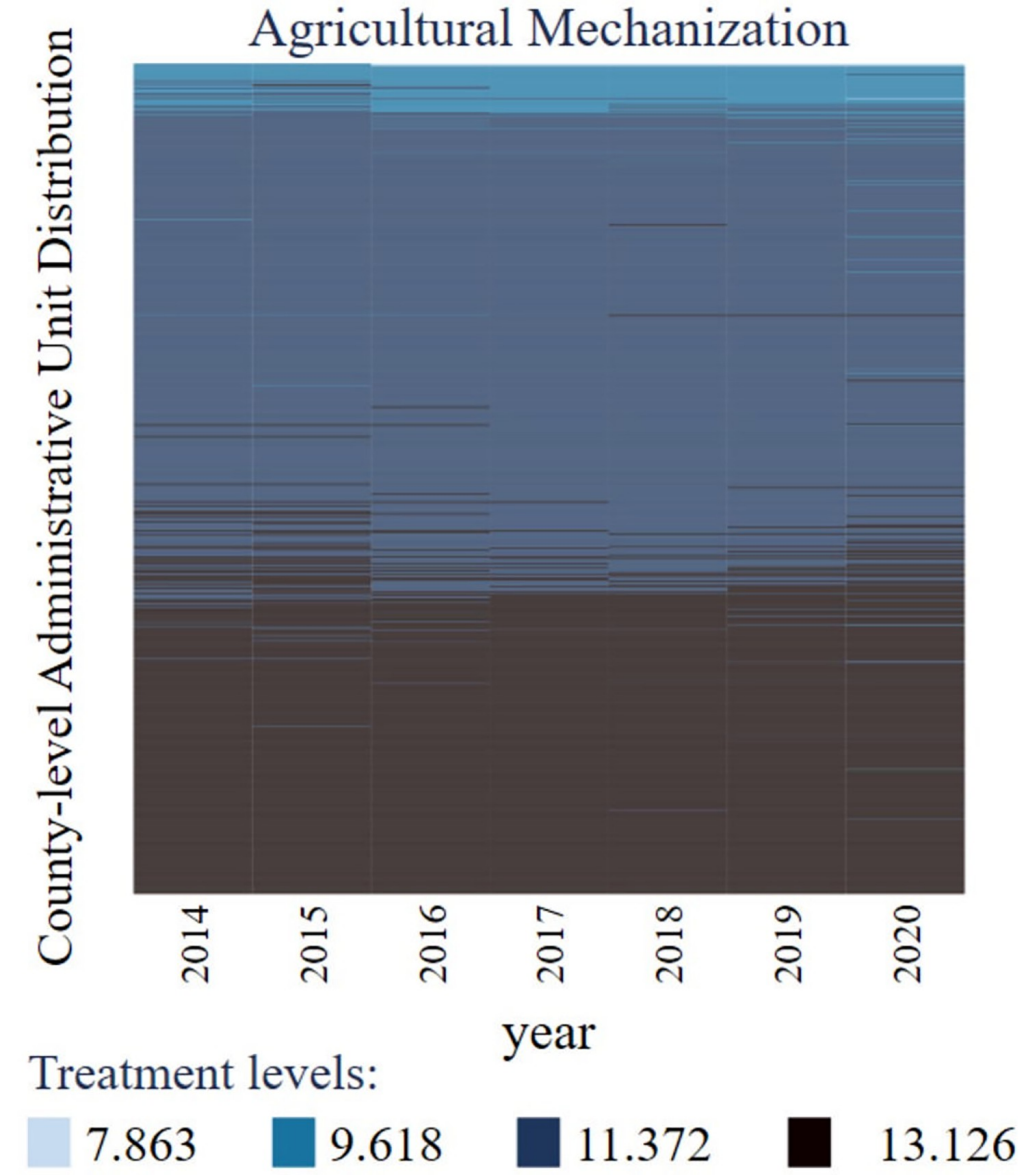
The visualization results of agricultural mechanization.

The explained variable is the level of agricultural mechanization. Regarding the studies of Li et al. (2018) [38], the total power of agricultural machinery was used to measure the level of agricultural mechanization. For smoothing the bias caused by endowment differences among counties, this paper takes the natural logarithm of the index as the explained variable (*ln*_*AgrMach*). The visualization results of agricultural mechanization level data are shown in Fig 2. The agricultural mechanization level is generally stable and evenly distributed after taking the logarithm. Besides, the missing value of agricultural mechanization level takes a small proportion, and the data has strong balance characteristics. From the data change trend, the level of agricultural mechanization is slowly improving but has prominent interval heterogeneity characteristics.

#### 3.2.2. Digital financial inclusion

**Fig 3:**
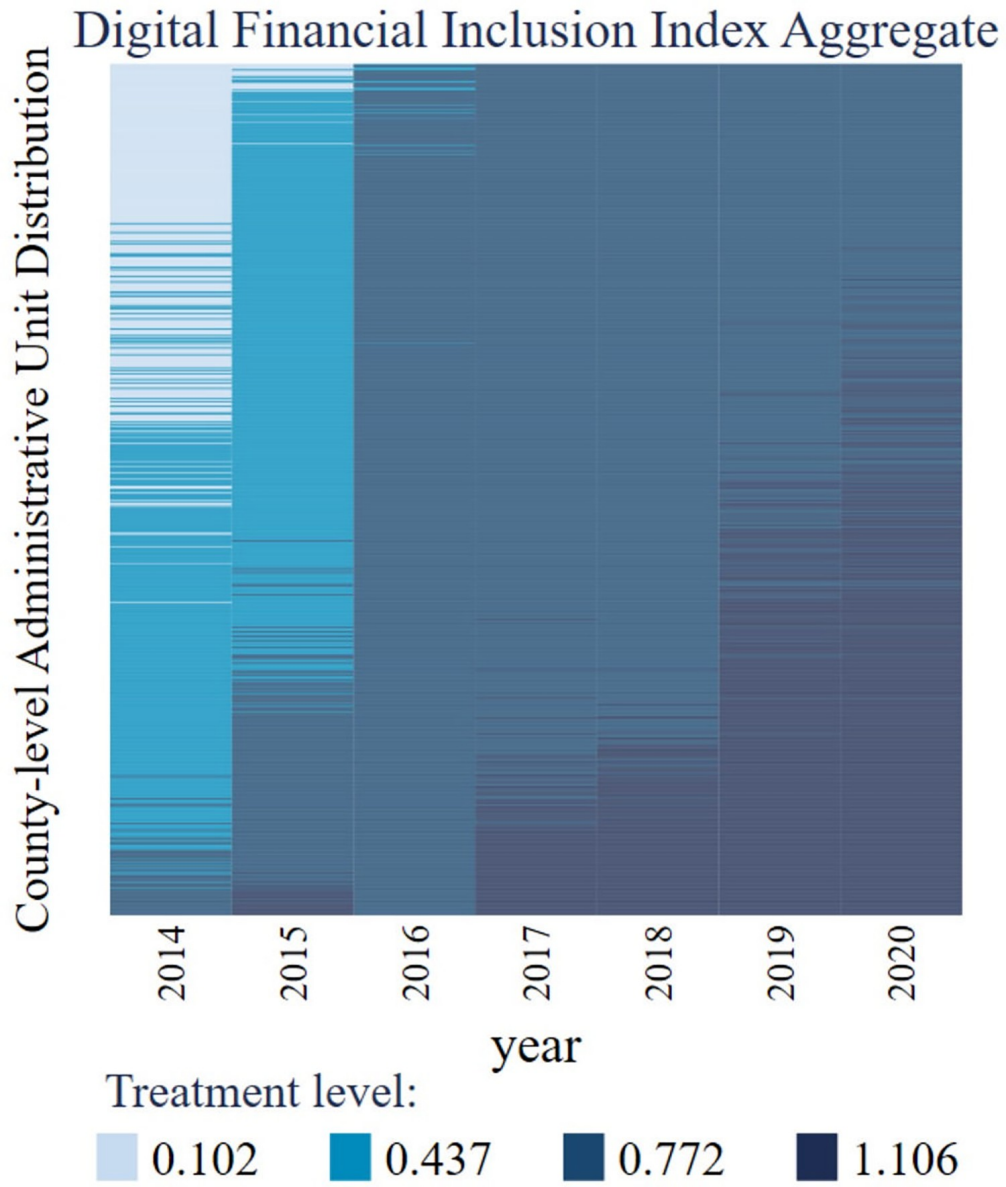
The visualization results of digital financial inclusion data.

This paper uses the county-level digital financial inclusion index as the explanatory variable to present the development of digital financial inclusion in China’s county-level administrative units. The paper uses the index aggregate as the core explanatory variable and threshold variable. The digital financial inclusion index aggregate (*DFI*_*ia*_) can be further divided into three dimensions: coverage breadth (*DFI*_*cb*_), usage depth (*DFI*_*ud*_), and digital level. The total index of digital inclusive finance is calculated as follows: the indexes with different properties and measurement units are treated without dimension, and then the weight is determined by the coefficient of variation method. Finally, the weight of the first-level index of coverage breadth is 54.0%, the weight of the first-level index of use depth is 29.7%, and the weight of the first-level index of digitalization degree is 16.3% [39]. Considering the weight of the dimensions and the specific influence of dimensions on agricultural mechanization, the analysis of sub-dimensions in this paper focuses on coverage breadth and usage depth. The digital financial inclusion index and its dimensions are divided by 100 in the model. In the robust test, the core explanatory variable is replaced by the logarithm of the digital financial inclusion index(ln_*DFI*_*ia*_). The visualization results of digital financial inclusion data are shown in Fig 3, and it can be found that the level of digital financial inclusion in counties of China is steadily improving. Besides, the missing value of digital financial inclusion takes a small proportion, and the data has strong balance characteristics.

#### 3.2.3. Urbanization

The paper uses the average nighttime light intensity of the region to measure the level of urbanization (*Urb*) in the area. Currently, most indicators for measuring urbanization use the proportion of urban population in the total population or the proportion of GDP in built-up areas to regional GDP. However, this single method of measuring population or economic proportion cannot fully reflect a region’s urbanization level. Unlike most countries, China’s urbanization is based on the registered residence registration system separating urban and rural areas. On the one hand, this has led to the inconsistency between the registered residence registration category and the actual population flow. Moreover, it has also caused long-term restrictions on population flow, leading to urbanization lagging behind industrialization. Although some scholars used the ratio of the urban permanent population in the current year to the total permanent population at the end of last year to optimize the measurement standard, the endogenous problem of mutual causality between population urbanization and agricultural mechanization still cannot be solved. If the method of measuring the proportion of GDP in built-up areas is adopted because China rarely counts the GDP of built-up areas at the county level, this will bring difficulties in measuring data. The urbanization indicators obtained from nighttime lighting data processing can solve the above problems effectively. As a representation of human activities, nighttime lighting can reflect social and economic factors such as population concentration and economic activity, thereby truly reflecting the urbanization status of a country or region [40–43].

#### 3.2.4. Control variables

The control variables include per capita GDP, financial dependence, financial agglomeration, the degree of local government fiscal strain, regional informatization level, and the amount of agricultural fertilizer applied. Use regional GDP divided by the total population of the region to represent GDP per capita (*GDP*_*per*_). Use general fiscal revenue of local government divided by the area GDP to represent financial dependence (*Fin*_*dep*_). Use the regional financial institutions’ loan balance at the end of the year divided by the regional GDP to represent financial agglomeration (*Fin*_*agg*_). Using regional fiscal revenue divided by regional fiscal expenditure to represent the degree of regional financial strain (*Degree*_*fs*_). Use the total amount of regional fixed telephones divided by the total population to represent the regional informatization level (*Inf*_*level*_). Using the amount of fertilizer used in agricultural production within the year after logarithmic treatment, including nitrogen, phosphate, potassium, and compound fertilizer to represent the amount of agricultural fertilizer applied (*ln*_*Arg*_*fer*_). The descriptive statistical results of all the above variables are shown in Table 1.

**Table 1:** Descriptive statistical analysis.

VARIABLES	(1)	(2)	(3)	(4)	(5)
	N	mean	sd	min	max
*ln*_*AgrMach*	42768	12.066	1.688	0	19.112
*DFI*_*ia*_	13767	0.917	0.238	0.102	1.441
*DFI*_*cb*_	13767	0.838	0.204	-0.235	1.748
*DFI*_*ud*_	13766	1.077	0.345	0.026	2.147
*DFI*_*dl*_	13766	0.889	0.301	-1.654	3.076
*Urb*	40761	1.003	4.643	0	78.668
*Fin*_*dep*_	38662	0.055	0.235	-6.186	25.014
*Degree*_*fs*_	39354	5.51	8.728	-21.479	737.1
*Inf*_*level*_	38614	0.135	0.192	-19.917	8.154
*GDP*_*per*_	44757	2.709	10.625	-818.49	1272.41
*Fin*_*agg*_	37698	0.602	0.923	-91.108	14.532
*ln*_*Arg*_*fer*_	34152	9.417	1.669	-3.912	13.878

### 3.3. Model

In terms of the selection of research methods, combined with the above theoretical analysis and the non-normal and large nature of the distribution of digital inclusive finance and agricultural mechanization in China’s counties, this paper adopts the ordinary least squares regression estimation method (OLS) to carry out econometric analysis on the influencing factors of agricultural mechanization. In addition, given the possible endogeneity problems and missing observed values of non-equilibrium panels, this paper selects the statistical regression model of standard error clustering to the county level as the baseline regression based on the two-way fixed effect.

The two-way fixed effect model can control both individual-fixed effects and time-fixed effects. The individual fixed effect can capture the influence of unobservable factors specific to the county dimension on the dependent variable, while the time fixed effect can capture the influence of unobservable factors specific to the time on the dependent variable. By controlling these fixed effects, we can reduce the bias of the estimated results of this model and provide more accurate inferences. It should be noted that the bidirectional fixed effect model can analyze data in multiple dimensions of individual and time, thereby utilizing more sample information and increasing the accuracy and efficiency of statistical inference, which is more suitable for the unbalanced panel data regression of 1309 Chinese counties in this paper. In addition, in panel data, there may be an endogenous relationship between variables, that is, a variable is both a dependent variable and an independent variable. The two-way fixed models can control this endogeneity through individual-fixed effects and time-fixed effects to provide more accurate estimates. Standard error clustering is a common error processing technique in panel data analysis. It takes the heterogeneity of panel data into account, eliminates the influence of heterogeneous errors, improves the effectiveness of statistical inference, and makes the model more adaptable to heterogeneous panel data structures.

The model estimation equation is set as follows:

$$ln\_AgrMach_{i,t}=\alpha +\beta \times DFI_{i,t}+\gamma \times Control+\mu_{i}+\varpi_{t}+\varepsilon_{i,t}$$ (1)

Where *ln*_*AgrMach*_*i*,*t*_ is the explained variable, which is the level of agricultural mechanization of *i* county in *t* year; *DFI*_*i*,*t*_ is the explanatory variable which is the digital financial inclusion index of *i* county in *t* year; *Control* represents control variables; *μ*_*i*_ represents entity fixed effects; *ϖ*_*t*_ represents time-fixed effect; *ε*_*i*,*t*_ represents stochastic error term.

In this paper, a mediation effect model is constructed to verify it. The mediation effect model constructed in this paper is shown as follows.


$$Urb=\alpha +\beta \times DFI+\gamma \times Control+\mu_{i}+\varpi_{t}+\varepsilon_{i,t}$$ (2)

$$ln\_AgrMach_{i,t}=\alpha +\beta \times DFI+\kappa \times Urb+\gamma \times Control+\mu_{i}+\varpi_{t}+\varepsilon_{i,t}$$ (3)

Where *Urb* is the mediating variable, which represents the level of urbanization.

## 4. Empirical results

### 4.1. Benchmark regression of impact of digital financial inclusion on agricultural mechanization

Based on the theoretical model and practical data, the relationship between county financial agglomeration, agricultural mechanization, and farmers’ income growth and their mutual transmission mechanism are deeply discussed. In this section, the random effect model, the bidirectional fixed effect model, and the bidirectional fixed and clustering to county method are used to estimate Eq (1), to investigate the total effect of digital inclusive finance on agricultural mechanization at the county level. The estimated results are shown in Table 2.

**Table 2:** Benchmark regression.

VARIABLES	(1)	(2)	(3)
	*ln*_*AgrMach*	*ln*_*AgrMach*	*ln*_*AgrMach*
*DFI*_*ia*_	-0.199***	0.297***	0.297***
	(0.032)	(0.055)	(0.076)
*GDP*_*per*_	-0.003	0.007***	0.007
	(0.002)	(0.002)	(0.006)
*Fin*_*dep*_	-1.873***	0.06	0.06
	(0.224)	(0.16)	(0.213)
*Fin*_*agg*_	-0.015	-0.013	-0.013
	(0.016)	(0.015)	(0.023)
*Degree*_*fs*_	-0.011***	0.004***	0.004***
	(0.001)	(0.001)	(0.001)
*Fin*_*agg*_	-0.330***	0.307***	0.307***
	(0.093)	(0.068)	(0.089)
*ln*_*Arg*_*fer*_	0.466***	0.174***	0.174***
	(0.006)	(0.013)	(0.035)
Observations	8209	8205	8205
R-squared	0.573	0.946	0.946
Year-fix	NO	YES	YES
Id-fix	NO	YES	YES
Cluster	NO	NO	YES

Column (1) of Table 2 shows the random effects model (RE), and the results show that county digital financial inclusion has a significant negative effect on agricultural mechanization, which is significant at the 1% significance level, after controlling for a series of other variables. This shows that the higher the level of digital financial inclusion, the more detrimental to the development of agricultural mechanization level. However, this result does not take into account the fact that individual unobservable heterogeneity intercepts are often correlated with explanatory variables or interfere with each other.

Therefore, after considering the influence of regional differences and time differences, the two-way fixed effect model (two-way FE) was adopted for estimation, and column (2) is the estimation result. The results of regression robust standard error clustering to counties based on bidirectional fixation are shown in column (3), and the two conclusions are similar. Column (3) was selected as the benchmark regression result. The results show that the county’s digital financial inclusion still has an impact on the growth of agricultural mechanization level, but it is no longer a negative stimulus effect but a positive effect, the correlation coefficient is 29.7%, which is significant at the significance level of 1%. This indicates that improving the level of digital financial inclusion is conducive to promoting the development of agricultural mechanization, and verifies hypothesis 1.

The possible explanations for this are as follows: First, due to the inclusive nature of digital inclusive finance, it not only provides capital elements for industry and service industry but also provides sufficient capital elements for the development of the agricultural economy. The increase in investment in agricultural capital elements is mainly reflected in the improvement of agricultural mechanization level in agriculture. Second, the digital inclusive financial platform represented by flower payment and cash loans improves individual digital skills in a convenient and unsecured way, lowers the financing threshold of the agricultural sector, and enables individual farmers to quickly obtain financial support from financial institutions, thus expanding the financing channels of the agricultural sector and ultimately promoting the improvement of agricultural mechanization. Third, the development of digital inclusive finance provides a guarantee for the construction of a digital economy platform, which provides an opportunity for the development of agricultural mechanization.

### 4.2. Endogeneity test of regression model

Considering the possibility of endogeneity in the above estimation, this paper adopts the instrumental variable method to carry out the endogeneity test. Referring to the practice of Zhou and Miao (2023), this paper chooses the one-period-lagged digital inclusive financial index (*l*.*DFI*_*i*,*t*_) as the instrumental variable for the endogeneity test [44], the instrumental variable model is set as Formula 4, and the regression results are shown in Table 3.


$$ln\_AgrMach_{i,t}=\alpha +\beta \times DFI_{i,t}+\gamma \times Control+\lambda \times l.DFI_{i,t}\times d.DFI_{i,t}+\mu_{i}+\varpi_{t}+\varepsilon_{i,t}$$ (4)

**Table 3:** Regression results of instrumental variables.

VARIABLES	(1)	(2)
	*DFI*_*ia*_	*ln*_*AgrMach*
*l*.*DFI*_*i*,*t*_	0.292***	
	(0.010)	
*DFI*_*ia*_		1.254***
		(0.285)
*GDP*_*per*_	-0.003***	0.012
	(0.001)	(0.009)
*Fin*_*dep*_	0.011	0.099
	(0.026)	(0.232)
*Fin*_*agg*_	0.002	0.005
	(0.003)	(0.023)
*Degree*_*fs*_	0.000**	0.004***
	(0.000)	(0.001)
*Fin*_*agg*_	0.064***	0.142
*ln*_*Arg*_*fer*_	(0.024)	(0.124)
	0.004	0.169***
	(0.003)	(0.044)
Kleibergen-Paap rk		328.184***
Cragg-Donald Wald F statistic		874.693
Kleibergen-Paap rk Wald F statistic		817.616
Observations	6,940	6,886
R-squared	0.940	0.569
yearfix	YES	YES
idfix	YES	YES

Instrumental variables reflect the level of county-level digital financial inclusion development with a lag of one period and can predict the growth of the digital financial inclusion level. The development level of county-level digital financial inclusion, which lags one stage behind, is exogenous to the agricultural mechanization level of county farmers and is related to the development of digital financial inclusion in the current period.

Column (1) is the regression result of the random effects model, column (2) is the regression result of the two-way fixed model, and (3) is the regression result of clustering standard errors to counties based on column (2). The P-value of the Kleibergen-Paaprk rkLN statistic of the unidentifiable test is 0.000. The relatively large values of the Cragg-Donald Wald F statistic and Kleibergen-Paap Wald rk F statistic indicate that the selection of instrumental variables in this paper is reasonable and meets the requirements of the exogeneity and correlation of instrumental variables.

There is a significant positive correlation between instrumental variables and digital inclusive finance, and digital inclusive finance has a significant positive impact on agricultural mechanization level. The estimated result is consistent with the baseline regression result, indicating that the above analysis conclusion is robust.

### 4.3. Mechanism analysis

This section uses the mediation effect test method to conduct an in-depth investigation on the intermediary effect of urbanization between the level of digital financial inclusion and the level of agricultural mechanization at the county level.

According to Eqs (2) and (3), the regression results are shown in Table 4. Among them, column (1) and column (3) are listed as considering the double-effect fixed effect, and column (2) and column (4) make the standard error cluster to the county area on the basis of the bidirectional fixed effect. According to Table 4, the mediating effect test of agricultural mechanization can be seen that the P-value of Z statistic of the Sobel test is less than 1% significance level, indicating that the null hypothesis is rejected, that is, the mediating effect of agricultural mechanization is significant.

**Table 4:** Mechanism test.

VARIABLES	(1)	(2)	(3)	(4)
	*Urb*	*Urb*	*ln*_*AgrMach*	*ln*_*AgrMach*
*DFI*_*ia*_	-0.537***	-0.537***	0.282***	0.282***
	(0.055)	(0.081)	(0.055)	(0.077)
*Urb*			-0.028**	-0.028**
			(0.012)	(0.013)
*GDP*_*per*_	0.050***	0.050***	0.008***	0.008
	(0.002)	(0.010)	(0.003)	(0.006)
*Fin*_*dep*_	0.724***	0.724**	0.080	0.080
	(0.162)	(0.312)	(0.160)	(0.213)
*Fin*_*agg*_	0.110***	0.110***	-0.010	-0.010
	(0.015)	(0.032)	(0.015)	(0.022)
*Degree*_*fs*_	-0.005***	-0.005***	0.003***	0.003**
	(0.001)	(0.001)	(0.001)	(0.001)
*Fin*_*agg*_	-1.076***	-1.076***	0.277***	0.277***
	(0.069)	(0.179)	(0.069)	(0.089)
*ln*_*Arg*_*fer*_	-0.081***	-0.081***	0.172***	0.172***
	(0.013)	(0.020)	(0.013)	(0.036)
Observations	8,256	8,256	8,205	8,205
R-squared	0.966	0.966	0.946	0.946
yearfix	YES	YES	YES	YES
idfix	YES	YES	YES	YES

According to Table 4, the correlation coefficient of digital inclusive finance on urbanization in columns (1) and (2) is -53.7%, which is significant at the 1% level. This indicates that the level of digital inclusive finance in counties has a significant and negative direct effect on urbanization. Digital divide and information asymmetry: Rural areas generally face the problem of digital divide and information asymmetry, and there may be insufficient technical equipment, digital education, and other problems. This may limit the positive effect of digital financial inclusion on urbanization. The existence of elite capture phenomenon is more likely to make the impact of digital inclusive finance on urbanization deviate from the original intention of inclusiveness. Empirical data regression results verify hypothesis H2a in this paper.

In columns (3) and (4), the coefficient of county-level digital inclusive finance is significantly positive, while the coefficient of urbanization variable is significantly negative, but the value is small. This indicates that after controlling the influence of the county-level financial agglomeration variable, the mediating variable of agricultural mechanization still has a significant effect on agricultural mechanization. This further verifies that agricultural mechanization plays an intermediary effect in the effect of farmers’ income growth of county financial agglomeration. The above empirical results show that urbanization plays an important conduction role in the influence path of digital inclusive finance → urbanization → agricultural mechanization level growth, which verifies the validity of hypothesis H2b and H2c in this paper.

According to induced technological change theory, rational farmers will increase relatively abundant and cheap production factor inputs to replace relatively scarce and expensive production factors. With the gradual disappearance of China’s demographic dividend, the agricultural labor force began to become a scarce element, and its price and opportunity cost of farming continued to rise. The process of urbanization will undoubtedly accelerate this phenomenon. Digital inclusive finance is conducive to reducing rural financial exclusion, easing farmers’ credit constraints, and reducing financing costs and agricultural production input costs. Rational farmers will increase financial capital and agricultural machinery capital input to replace labor factors, and financial capital input will accelerate the advancement of agricultural mechanization operations. Therefore, digital inclusive finance exerts an indirect effect on urbanization and reduces the level of agricultural mechanization by curbing the urbanization process. However, the direct effect of digital inclusive finance on the level of agricultural mechanization is significantly positive, so the overall effect results in promoting the development of agricultural mechanization level.

### 4.4. Robust test

#### 4.4.1. Replacing explanatory variables

In the previous discussion on the impact of digital inclusive finance on agricultural mechanization, the county digital inclusive finance index was divided by 100 as the core explanatory variable. This part attempts to study the impact of digital financial inclusion on agricultural mechanization with the natural logarithm of the digital financial inclusion index as the core explanatory variable. As shown in column (1) of Table 5, the natural logarithm of digital inclusive finance has a significant positive impact on the level of agricultural mechanization, which is consistent with the previous results, indicating that the estimated results of the impact of digital inclusive finance on agricultural mechanization are stable.

**Table 5:** Robustness test results.

VARIABLES	(1)	(2)	(3)	(4)
	*ln*_*AgrMach*	*ln*_*AgrMach*	*ln*_*AgrMach*	*ln*_*AgrM*_*per*_
*ln*_*DFI*_*ia*_	0.162***			
	(0.035)			
*DFI*_*ia*_		0.379**	0.299***	0.177**
		(0.156)	(0.083)	(0.084)
*Urb*	-0.024*	0.02	-0.006	-0.082***
	(0.013)	(0.039)	(0.014)	(0.016)
*GDP*_*per*_	0.009	0.023	0.01	0.057***
	(0.006)	(0.019)	(0.007)	(0.008)
*Fin*_*dep*_	0.041	0.521	0.206	0.625**
	(0.213)	(0.446)	(0.231)	(0.260)
*Fin*_*agg*_	-0.01	-0.006	-0.015	0.041
	(0.022)	(0.037)	(0.023)	(0.026)
*Degree*_*fs*_	0.003**	0.011***	0.006***	0.006***
	(0.001)	(0.004)	(0.002)	(0.002)
*Fin*_*agg*_	0.260***	0.295	0.250**	1.060***
	(0.089)	(0.217)	(0.099)	(0.149)
*ln*_*Arg*_*fer*_	0.171***	0.168***	0.171***	0.177***
	(0.036)	(0.055)	(0.038)	(0.035)
Observations	8205	8256	8256	8205
R-squared	0.946	0.785	0.928	0.89
yearfix	YES	YES	YES	YES
idfix	YES	YES	YES	YES

#### 4.4.2. Winsorization

In order to eliminate the impact of extreme values on research, continuous variables are generally subjected to winsorization. Firstly, winsorize the data by 1% up and down which means for numbers less than 1%, assign a value of 1%, and for numbers greater than 99%, assign a value of 99%. The regression results are shown in column (2). Secondly, winsorize the data by 5% up and down which means for numbers less than 5%, assign a value of 5%, and for numbers greater than 95%, assign a value of 95% as the regression results shown in the (3) column of Table 5. The results of regression are consistent with the baseline regression, which proves the robustness of the baseline regression.

#### 4.4.3. Replacing explained variables

The explained variable is replaced with the total power of agricultural machinery per capita and logarithm. The specific calculation method is the total power of agricultural machinery in a county divided by the total population in a county, then take logarithm of the final value. The specific correlation coefficient is shown in column (4). The positive promoting effect of digital inclusive finance has been reduced, while the negative restraining effect of urbanization has been further amplified. However, the direction and numerical value are consistent with the previous analysis results on the whole, which proves the robustness of benchmark regression and mechanism analysis.

### 4.5. Coverage breadth and usage depth of DFI

Digital financial inclusion coverage breadth is the scope and breadth of digital financial services in terms of popularization and coverage, the core index is account coverage, including the number of Alipay accounts per 10,000 people, the proportion of Alipay cardbound users and the average number of bank cards bound to each Alipay account. The usage depth of digital financial inclusion refers to the actual use of digital financial services. Specifically, in terms of financial services, it includes payment services, currency based financial services, credit services, insurance services, investment services, and credit services. From the perspective of usage, it includes both the actual total use index (the number of users per 10,000 Alipay users who use these services) and the active use index (the number of transactions per person and the amount of transactions per person) [39].

**Fig 4:**
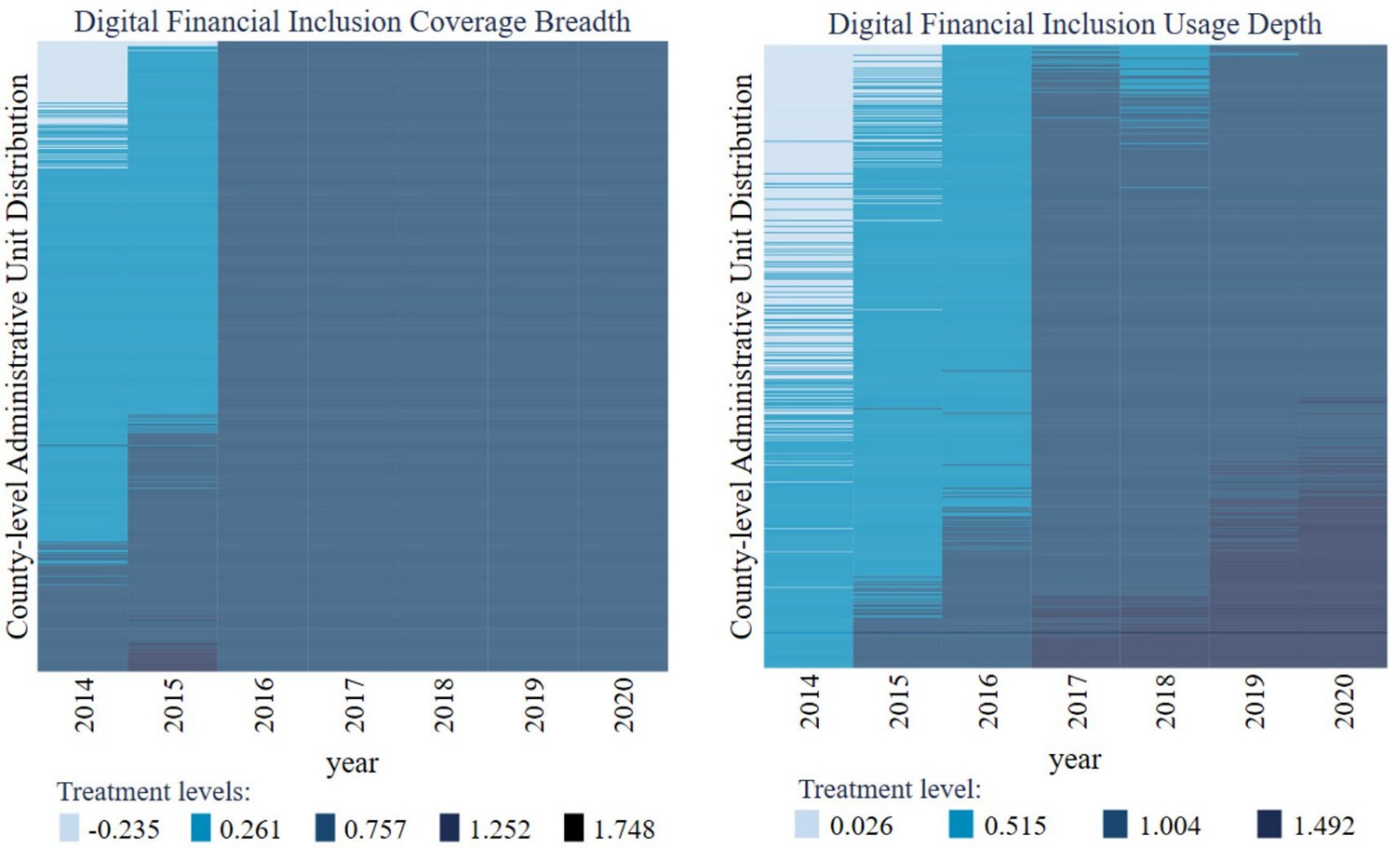
The visualization results of the coverage breadth and usage depth data.

The visualization of data distribution of the coverage breadth and usage depth of digital financial inclusion is shown in Fig 4. The missing value of the usage depth and coverage breadth of digital inclusive finance accounts for a small proportion, almost negligible. In addition, the coverage breadth of digital inclusive finance is increasing rapidly year by year. While the usage depth of digital inclusive finance has a certain volatility, but it shows an increasing trend on the whole.

Using the two-way fixed effect model and clustering the standard error to the county, the regression results are shown in columns (1)—(2) of Table 6. The coverage breadth and usage depth of digital inclusive finance have a significant positive correlation coefficient on agricultural mechanization, which means that from the perspective of the coverage breadth and usage depth, digital inclusive finance has a significant positive effect on the level of military agricultural mechanization. This is consistent with the result of baseline regression, which further proves the robustness of baseline regression.

**Table 6:** Regression results of the coverage breadth and usage depth.

VARIABLES	(1)	(2)
	*ln*_*AgrMach*	*ln*_*AgrMach*
*DFI*_*cb*_	0.244***	
	(0.051)	
*DFI*_*ud*_		-0.288***
		(0.069)
*Urb*	-0.018	-0.030**
	(0.013)	(0.013)
*GDP*_*per*_	0.009	0.007
	(0.006)	(0.006)
*Fin*_*dep*_	0.042	0.090
	(0.213)	(0.212)
*Fin*_*agg*_	-0.013	-0.013
	(0.022)	(0.022)
*Degree*_*fs*_	0.003**	0.004***
	(0.001)	(0.001)
*Fin*_*agg*_	0.260***	0.315***
	(0.089)	(0.090)
*ln*_*Arg*_*fer*_	0.170***	0.171***
	(0.036)	(0.035)
Observations	8,205	8,204
R-squared	0.946	0.946
yearfix	YES	YES
idfix	YES	YES

### 4.6. Heterogeneity test

Considering the heterogeneity of resource endowments at county level in China, this paper will further investigate the heterogeneity of the contents of county digital inclusive programs and urbanization on the improvement of agricultural mechanization.

The samples were divided into two sub-samples according to the main grain production areas and grain production balance areas, and the regression was carried out respectively. The results are shown in Table 7 column (1) and (2). The correlation coefficient of digital financial inclusion on the level of agricultural mechanization in the grain production balance area is much greater than that in the main grain-producing area, which indicates that the positive stimulating effect of digital financial inclusion on the level of agricultural mechanization in the grain production balance area is much greater than that in other areas. The possible explanation for this is that the agricultural production base of the main grain producing areas is good, and the existing level of mechanization is relatively high and the room for improvement is small. The grain production balance area conforms to the law of backward and catch-up, because the level of agricultural mechanization is backward compared with the other, so the progress space is huge and the speed of improvement is faster.

**Table 7:** Regression results of heterogeneity analysis.

VARIABLES	(1)	(2)	(3)	(4)	(5)	(6)
	*ln*_*AgrMach*	*ln*_*AgrMach*	*ln*_*AgrMach*	*ln*_*AgrMach*	*ln*_*AgrMach*	*ln*_*AgrMach*
*DFI*_*ia*_	0.280***	0.661***	0.359**	0.254***	0.244*	0.405***
	(0.086)	(0.176)	(0.179)	(0.083)	(0.126)	(0.141)
*Urb*	-0.044***	-0.02	-0.140**	-0.009	-0.007	-0.166**
	(0.015)	(0.040)	(0.061)	(0.014)	(0.014)	(0.072)
*GDP*_*per*_	0.008	0.011	0.006	0.009	0.013	-0.001
	(0.008)	(0.008)	(0.014)	(0.007)	(0.009)	(0.008)
*Fin*_*dep*_	0.195	-0.346	-0.060	0.059	0.019	-0.162
	(0.269)	(0.359)	(0.605)	(0.222)	(0.272)	(0.323)
*Fin*_*agg*_	-0.038	0.038	-0.100**	0.026	0.032	-0.020
	(0.030)	(0.039)	(0.040)	(0.024)	(0.057)	(0.022)
*Degree*_*fs*_	0.005***	0.004**	0.002	0.004**	0.005	0.002
	(0.002)	(0.002)	(0.002)	(0.002)	(0.004)	(0.001)
*Fin*_*agg*_	0.162*	0.507***	0.314*	0.242**	0.204*	0.379**
	(0.096)	(0.173)	(0.182)	(0.101)	(0.109)	(0.147)
*ln_Arg*_*fer*_	0.221***	0.058**	0.064**	0.232***	0.191***	0.095***
	(0.042)	(0.027)	(0.026)	(0.043)	(0.047)	(0.031)
Observations	5499	2706	2195	6010	3877	4154
R-squared	0.949	0.925	0.941	0.948	0.946	0.956
yearfix	YES	YES	YES	YES	YES	YES
idfix	YES	YES	YES	YES	YES	YES

The samples were divided into two sub-samples according to whether they were the national-level poor county or contiguous areas of dire poverty in China, and the regression was carried out respectively. The results are shown in Table 7 column (3) and (4). The correlation coefficient of digital financial inclusion in samples from poor counties is higher than that of non-poor counties, which indicates that digital financial inclusion has a greater role in promoting agricultural mechanization in poor counties. The possible explanation for this is that according to the theory of diminishing marginal return on capital in neoclassical economic theory, relatively backward regions will achieve "catch-up development" and are more likely to rely on inclined financial resource investment and inclusive financial policies, and take advantage of their late-comer advantages to achieve additional agricultural mechanization level improvement.

According to the median of the digital financial inclusion level year by year, the sample was divided into two sub-samples above and below the median, and the regression was carried out respectively, as shown in Table 7 column (5) and (6). The correlation coefficient of samples with relatively high development level and foundation of digital financial inclusion is much higher than that of regions with relatively backward digital financial inclusion level. This shows that in areas where digital inclusive finance is well developed, it is easier for digital inclusive finance to play a leverage role and further amplify the role of improving the level of agricultural mechanization. The possible explanation for this is that where the foundation for digital financial inclusion is better, the penetration of digital channels such as smartphones and the Internet is higher, and it is easier for people to access and use digital financial services. This allows digital financial inclusion to cover users more widely and provide more diversified and convenient financial products and services. At the same time, these regions usually have a more complete digital financial ecosystem, including digital payment platforms, fintech companies, financial institutions, etc. The development of these institutions and platforms can provide more diversified digital financial products and services to meet the different financial needs of users.

## 5. Conclusions and policy insight

From the perspective of county-level digital inclusive finance, this paper expounds on the influence mechanism between the level of urbanization and agricultural mechanization, forms a research hypothesis, and then examines the total effect of county-level digital inclusive finance on the growth of agricultural mechanization based on the unbalanced panel data of 1309 counties in China from 2014 to 2020. At the same time, it also studies the transmission mechanism of digital inclusive finance at the county level affecting the level of agricultural machinery through urbanization. Through the heterogeneity analysis, we studied the impact of digital inclusive finance and urbanization on the level of agricultural mechanization by different resource endowments and attributes of different counties in China.

This paper found that, first, the county’s digital financial inclusion has a significant positive effect on the growth of the agricultural mechanization level. Second, digital inclusive finance at the county level can also indirectly affect the growth of agricultural mechanization through urbanization, that is, agricultural mechanization has an intermediary effect between the financial agglomeration at the county level and the growth of farmers’ income. Third, the impact of county-level digital financial inclusion on the growth of agricultural mechanization level is significantly heterogeneous, and the promoting effect will be significantly increased in areas such as balanced grain production, national-level poor county or contiguous areas of dire poverty, and areas with a good foundation for digital financial inclusion. Based on the above conclusions, this paper puts forward the following three policy recommendations.

First, the government can further promote agricultural mechanization through the development of digital financial inclusion. Digital inclusive finance is a new path for agricultural mechanization, and efforts should be made to improve the ability of digital inclusive finance to serve agriculture and rural areas. Develop digital inclusive financial service models that are more in line with rural characteristics, to better promote digital inclusive finance to improve agricultural mechanization.

Second, the government should guide and accelerate the process of digital financial inclusion promoting urbanization, thereby amplifying the positive impact of digital financial inclusion on agricultural mechanization. As an important intermediate variable for digital inclusive finance to improve the level of agricultural mechanization, urbanization should be guided by special government policies to avoid negative effects on urbanization caused by the exclusion of financial attributes and elite capture of digital inclusive finance.

Third, because of the heterogeneity of the impact of digital financial inclusion on agricultural mechanization, local development should pay attention to different dimensions of digital financial inclusion according to specific conditions. For regions with a relatively backward social and economic foundation, attention should be paid to enlarging the inclusion of digital inclusive finance, and in contrast, in regions with a high level of digital inclusive finance development, attention should be paid to enlarging its financial leverage, to better promote the development of agricultural mechanization.

The findings of this paper are not only applicable to China but also to rural areas in other developing countries similar to rural China, especially those remote areas that are vulnerable but dependent on agriculture. Promoting agriculture through the inclusion of digital financial inclusion is an effective way to improve agricultural development in these regions and increase agricultural production efficiency and output. It is worth noting that there are some shortcomings in this study. First of all, due to the limited sources and availability of county dimension data in China, the data in this paper only covers 2014–2020, and other scholars can carry out relevant research on different dimensions based on the updated data in the future. Secondly, to better preserve the integrity and authenticity of the data, this paper directly conducts regression analysis on the unbalanced panel based on the bidirectional fixed model, so that the autocorrelation of spatial dimensions cannot be measured. In the future quantitative development process, there may be better econometric models that can use non-equilibrium panels, taking into account individual and time-fixed effects while taking into account geographical proximity and interaction.

## Supporting information

S1 Data Note: PKTKDQ: if the county is the national-level poor county or contiguous areas of dire poverty, PKTKDQ = 1, else PKTKDQ = 0; LSZCQ: if the county is the main grain producing areas, LSZCQ = 1; if the county is the grain production balance areas, LSZCQ = 2; Urb_average nighttime light intensity of the region; AgrMachPower_2: the amount of the total power of agricultural machinery; FertilizerConsm_2: the amount of fertilizer used in agricultural production within the year; L4: *Fin*_*dep*_, financial dependence; L5: *Degree*_*fs*_, degree of regional financial strain; K1: *Inf*_*level*_, regional informatization level; L2_wan: *GDP*_*per*_, GDP per capita; L6_w: *Fin*_*agg*_, financial agglomeration; aggregate_baifenyi: *DFI*_*ia*_, the index aggregate of digital financial inclusion index/100; coverage_breadth_baifenyi: *DFI*_*cb*_, the coverage breadth of digital financial inclusion index/100; usage_depth_baifenyi: *DFI*_*ud*_, the usage depth of digital financial inclusion index/100; digitization_level_baifenyi: the digital level of digital financial inclusion index/100; D_group(id). (DTA)
